# Differences in Circulating microRNAs between Grazing and Grain-Fed Wagyu Cattle Are Associated with Altered Expression of Intramuscular microRNA, the Potential Target *PTEN*, and Lipogenic Genes

**DOI:** 10.1371/journal.pone.0162496

**Published:** 2016-09-09

**Authors:** Susumu Muroya, Masahiro Shibata, Masayuki Hayashi, Mika Oe, Koichi Ojima

**Affiliations:** 1 Animal Products Research Division, NARO Institute of Livestock and Grassland Science, Tsukuba, Ibaraki, Japan; 2 Livestock Production and Wildlife Management Research Division, NARO Western Region Agricultural Center, Ohda, Shimane, Japan; 3 Animal Physiology and Nutrition Research Division, NARO Institute of Livestock and Grassland Science, Tsukuba, Ibaraki, Japan; University of Minnesota Medical Center, UNITED STATES

## Abstract

We aimed to understand the roles of miRNAs in the muscle tissue maturation and those of circulating microRNAs (c-miRNAs) in beef production of Japanese Black (JB) cattle (Wagyu), a breed with genetically background of superior intermuscular fat depot, by comparing different feeding conditions (indoor grain-feeding vs. grazing on pasture). The cattle at 18 months old were assigned to pasture feeding or conventional indoor grain feeding conditions for 5 months. Microarray analysis of c-miRNAs from the plasma extracellular vesicles led to the detection of a total of 202 bovine miRNAs in the plasma, including 15 miRNAs that differed between the feeding conditions. Validation of the microarray results by qPCR showed that the circulating miR-10b level in the grazing cattle was upregulated compared to that of the grain-fed cattle. In contrast, the levels of miR-17-5p, miR-19b, miR-29b, miR-30b-5p, miR-98, miR-142-5p, miR-301a, miR-374b, miR-425-5p, and miR-652 were lower in the grazing cattle than in the grain-fed cattle. Bioinformatic analysis indicated that the predicted target genes of those c-miRNAs were enriched in gene ontology terms associated with blood vessel morphogenesis, plasma membrane, focal adhesion, endocytosis, collagen, ECM-receptor interaction, and phosphorylation. In the grazing cattle, the elevation of miR-10b expression in the plasma was coincident with its elevation in the *longissimus lumborum* (LL) muscle. Expression of bovine-specific miR-2478, the most plasma-enriched miRNA, tended to be also upregulated in the muscle but not in the plasma. Furthermore, grazing caused the downregulated mRNA expression of predicted miR-10b and/or miR-2478 target genes, such as *DNAJB2*, *PTEN*, and *SCD1*. Thus, the feeding system used for JB cattle affected the c-miRNAs that could be indicators of grain feeding. Among these, miR-10b expression was especially associated with feeding-induced changes and with the expression of the potential target genes responsible for glucose homeostasis and intramuscular fat depot in the LL muscle of JB cattle.

## Introduction

MicroRNAs (miRNAs) are highly conserved, noncoding small RNAs that regulate the expression of target genes in various biological processes in plants and animals [1]. The mature miRNA is generated by processing from the initial transcript (pri-miRNA) via pre-miRNA and recognizes its target genes as a component of the RNA-induced silencing complex (RISC), resulting in mRNA degradation or destabilization. Approximately 30% of protein-coding genes are under the regulation of miRNAs [2].

To better understand the meat quality traits of domestic animals, skeletal muscle miRNAs have been profiled in investigations into how distinct muscle properties are specified. Some muscle miRNAs are differently expressed between muscle types [3,4] or tissues [5,6] in cattle and pigs. Notably, we have demonstrated that miR-196a/b and miR-885 in cattle are expressed exclusively in fast-glycolytic *semitendinosus* (ST) muscle but not in slow-oxidative *masseter* muscle [4]. In addition, skeletal muscle miR-206 and miR-208b expression is temporally upregulated, accompanying the downregulation of myosin heavy chain 2x expression, in grazing Japanese Shorthorn (JSH) cattle whereas the expression did not change in housed [7]. Since muscle physiological property determined by the muscle type have impacts on meat quality [8–10], potential association of miRNAs with muscle gene expression and mat quality have been focused recently [11–13]. Besides skeletal muscle, transcriptomic miRNA analyses in mammalian adipose tissue have unveiled their roles in fat accumulation. The relevance of miRNAs has also been pronounced in subcutaneous and/or visceral fat accumulation in response to growth and dietary nutrition in mice [14–16], sheep [17], and cattle [18]. When primary cultured porcine adipocytes are exposed to miR-130b-enriched micro-vesicles, peroxisome proliferator-activated receptor γ (*PPARG*) expression is downregulated [19].

Cattle feeding systems and dietary nutrients influence the physiological and histological muscle properties as well as growth performance. Compared to the muscles of housed cattle, those of grazing cattle potentially have distinct physiological properties and chemical compositions, including fatty acids and muscle proteins, due to the intermittent movements involved in grazing, which results in changes in subsequent beef quality. Japanese Black (JB; original cattle breed of Wagyu) cattle are conventionally fed a grain-rich concentrate in cattle houses in order to promote their genetically superior intramuscular fat (IMF) depot, which makes beef tender and gives JB beef the high marbling score that is so prized. Thus, differences in muscle growth and IMF deposition resulting from different feeding systems have been progressively investigated.

In particular, the molecular mechanisms underlying IMF depot in different feeding conditions, including diet composition, have been focused on for adipogenic and myogenic gene expression [20,21]. The *longissimus lumborum* (LL) and ST muscles of pasture-feeding JB cattle show lower extractable lipid content and expression of adipogenic *PPARG* and CCAAT-enhancer binding protein α (*CEBPA*) than those of housed JB cattle [22]. The expression patterns of *PPARG* and *CEBPA* in intramuscular adipose tissue are higher in cattle fed a high concentrate than those fed a low concentrate [21], indicating that the dietary roughage/concentrate ratio affects IMF fat depot-specific differences in adipogenic gene expression. It is therefore tempting to hypothesize that influences of dietary and feeding conditions on myogenic and adipogenic gene expression are regulated by miRNA, as suggested by differences in miRNAs between subcutaneous and visceral fat [18]. Understanding potential roles of miRNAs in muscle and adipose tissue development will provide new insights into the molecular basis of beef production and meat quality performance.

Moreover, circulating miRNAs (c-miRNAs), which are considered as potential indicators of disease and physiological conditions, also are potentially associated with muscle physiology and gene expression as suggested previously [23,24]. Likewise, use of c-miRNA as a monitoring marker could contribute to management and improvement of better beef production. Nevertheless, influences of dietary and feeding conditions on c-miRNAs in beef cattle have never been investigated except for effect of grazing of cattle [24].

In the present study, we therefore aimed to determine (1) the c-miRNAs that are affected by differences between grazing and indoor grain feeding of JB cattle, using an extracellular vesicle (EV)-preparation method from plasma [24], and (2) the muscle miRNAs involved in the feeding-induced regulation of beef quality-related gene expression. The grain-feeding condition employed here leads to higher IMF depot in the JB cattle muscle than the grazing condition [22]. Furthermore we discuss how the c-miRNA changes are associated with skeletal muscle miRNA expression, to understand potential impact of both muscle and circulating miRNAs on beef quality-related gene expression of JB cattle.

## Materials and Methods

### Animals

The animals were cared for as outlined in the Animal Experimental Guidelines of the NARO Western Region Agricultural Research Center (NARO/WARC) established by the Animal Care Committee, NARO/WARC, and this committee approved the study. All efforts were made to minimize suffering. Eight 10-month-old JB steers, bred at NARO/WARC, were grain-fed separately in a stall barn with no weight difference among the cattle until 10 months of age. Then they were fed a concentrate diet (flaked corn, flaked barley, wheat bran, and soybean meal; 73% total digestible nutrients (TDN) and 11.0% crude protein (CP) on a dry matter (DM) basis) *ad libitum* and 1.5 kg grass hay (43% TDN and 6.3% CP on a dry matter basis) until 16 months of age. Beginning at 18 months of age, the steers were divided into two groups for 5 months: 4 were continually fed the concentrate diet and grass hay in house and the other 4 were allocated to graze on Italian ryegrass pasture. The health of the animals was monitored every day, which satisfied the criteria of animal welfare defined in the Animal Experimental Guidelines of NARO/WARC. The grass mass was measured during the grazing period and their chemical composition was analyzed (Agricultural Chemistry Research Institute, Hokkaido, Japan) (Table 1). Body weight after the 5-month grazing period differed between the grain-fed (675.5 kg) and the grazing (571.5 kg) cattle (*P* = 0.030).

**Table 1:** Chemical composition of grass on pasture during grazing of cattle.

Grass mass (kg of DM/ ha)	TDN (% of DM)	CP (% of DM)	ADF (% of DM)	NDF (% of DM)
3,787	72.7	14.9	22.7	48

DM: dry matter, TDN: total digestible nutrients, CP: crude protein, ADF: acidic detergent fiber, NDF: neutral detergent fiber.

### Sample collection

A blood sample was drawn from the jugular vein of each animal at the age of 22 months, and the plasma was prepared with 0.1% EDTA followed by storage at -80°C until use. The LL samples were taken from by biopsy at 22 months. The biopsy procedure was as follows: the animal was locally anesthetized by an intramuscular injection of xylazine (Bayer, Tokyo, Japan) and a subcutaneous injection of lidocaine (AstraZeneca, Osaka, Japan). An incision was subsequently made in the skin overlying the LL muscle [25]. All samples were rapidly frozen in liquid nitrogen and stored at -80°C until RNA extraction.

### Plasma sample processing

We previously succeeded in preparing miRNA-enriched EVs as exosomes [24]. In the present study we used the same method to prepare miRNA-containing EVs from plasma. In brief, 10 ml of the plasma sample was mixed with 20 ml of PBS and centrifuged at 1,200 *g*, 4°C for 20 min. The supernatant was centrifuged at 12,000 *g*, 4°C for 45 min and further centrifuged at 110,000 *g*, 4°C for 120 min. The precipitation was suspended in PBS and centrifuged at 110,000 *g*, 4°C for 70 min. The final precipitation was resuspended in PBS, stored at 4°C for a few days, and then processed for RNA preparation.

### RNA preparation

Total RNA including miRNA was extracted from muscle or plasma precipitate using the mirVana microRNA isolation kit (Life Technologies Japan Ltd., Tokyo, Japan) for microarray analysis of plasma samples according to the manufacturer’s protocols. The quantity and quality of the RNA were confirmed by an Agilent Bioanalyzer 2100 with an RNA 6000 Pico Kit (Agilent Technologies, Santa Clara, CA, USA) before the RNA was applied to microarray analysis. Muscle total RNAs for qPCR analysis of muscular mRNA expression were prepared using ISOGEN (Nippon Gene, Tokyo, Japan).

### Microarray analysis

Of the four plasma precipitate samples, we used the three samples that satisfied the quantitative requirement as microarray samples for each cattle group. The plasma EV RNA samples for each feeding treatment (grazing or indoor grain-fed) were applied to a custom SurePrint G3 8x60K microarray (Agilent Technologies) that was designed using miRBase ver. 21 (Design ID: 077173). The miRNA Microarray System with miRNA Complete Labeling and Hybridization Kit (Agilent Technologies) was used according to the manufacturer’s protocol. The Agilent microRNA Spike-In Kit was used for in-process control to measure labeling and hybridization efficiency. The labeled samples were hybridized with the probes on the custom microarray, and then the arrays were scanned on a G4900DA SureScan scanner (Agilent Technologies) at 3 μm resolution. Microarray signals were extracted using Agilent Feature Extraction software v11.0 (Agilent Technologies), and the signal values were globally normalized to the 90 percentile using GeneSpring GX (Agilent Technologies), as we did previously to successfully analyze plasma exosomal miRNA samples [24].

To determine significantly different miRNAs between the groups, the normalized signal values were further applied to statistical analysis by significance analysis of microarrays (SAM) [26]. The data were computed with R 2.13.1 (http://cran.r-project.org). The miRNAs were considered significantly different between the groups if the resulting *P*-value < 0.05. Array data were deposited in the National Center for Biotechnology Information (NCBI) Gene Expression Omnibus (GEO) database, and are accessible through GEO Series accession number GSE81946 (http://www.ncbi.nlm.nih.gov/geo).

### cDNA synthesis

cDNA was synthesized from 250 ng of total RNA for muscle samples or 9 μl of the final product of RNA preparation for EV samples, using the miScript II RT kit (Qiagen, Tokyo, Japan) at 37°C for 60 min, and then the enzyme was inactivated at 95°C for 5 min. The cDNAs for muscle samples were each synthesized from 1,000 ng of total RNA by the ReverTra Ace qPCR RT kit (Toyobo, Tokyo, Japan).

### Quantitative PCR (qPCR) analysis

qPCR was performed using the CFX96 thermal cycler (Bio-Rad, Hercules, CA, USA) under the following program: 15 min at 95°C, followed by 40 cycles of 15 s at 95°C and 30 s at 60°C. For both plasma and muscle samples, miRNA qPCR was performed using the Thunderbird SYBR qPCR kit (Toyobo) in combination with the miScript Primer Assay for let-7g, RNU6-6P RNA (RNU6-6P), miR-10b, miR-19a, miR-21-5p, miR-26a, miR-27b, miR-29b, miR-30b-5p, miR-30d-5p, miR-92a, miR-98, miR-103, miR-142-5p, miR-144, miR-196a, miR-197, miR-199-3p, miR-204, miR-206, miR-208b, miR-301a, miR-338, miR-345-5p, miR-374b, miR-378, miR-381, miR-425-5p, miR-451, miR-486, miR-499, miR-652, miR-885, miR-2412, and miR-2478 (Qiagen) according to the manufacturer’s protocol. The qPCR analysis of elongase of very long chain fatty acids 6 (*ELOVL6*), stearoyl-Coenzyme A desaturase 1 (*SCD1*), phosphatase and tensin homolog (*PTEN*), profilin 2 (*PFN2*), DnaJ heat shock protein family (Hsp40) member B2 (*DNAJB2*), and *PPARG2* was performed using the Thunderbird SYBR qPCR kit (Toyobo), followed by normalization with the expression of ribosomal protein L7 (*RPL7*; an internal control) [4]. The sequences of the primers used are shown in Table 2. Differences between the groups in the expression ratios of the target miRNA/let-7g for plasma samples, the target miRNA/RNU6 for muscle samples, and that of mRNA/*RPL7* were compared. Melting curve analysis was used to confirm the specificity of the amplification reactions.

**Table 2:** PCR primer sequences of mRNAs targeted in this study.

Gene	Accession	Sequence		Product size (bp)
		Forward	Reverse	
*COL3A1*	NM_001076831.1	GGGACCTGGTTACTTTTCGCTC	TCAGGGTTGGGGCAGTCTAAT	212
*DNAJB2*	NM_001034592.1	TAGACGTACCGCGAAGTGC	TCTCACGCTTATGCTTGTCGG	169
*ELOVL6*	BC148954.1	AGCACCCGAACTAGGAGATACAAT	TACCAGGAGTACAGAAGCACAGTGA	102
*PFN2*	NM_001128197.1	GGGCGTCTTCCAGAGCATTA	GTCACCGTCCACGTATAGGC	139
*PPARG2*	NM_181024.2	TATTCTCAGTGGAGACCGCC	CTGCACGTGTTCTGTCACAAT	181
*PTEN*	NM_001319898.1	TTCCTGCAGAAAGACTTGAAGGT	AACTCTGCAATTAAATTTGGCGGT	146
*SCD1*	NM_173959.4	ACCTGGCTGGTGAATAGTGC	AAGTTGATGTGCCAGCGGTA	176

### Prediction and functional annotation of miRNA target genes

The miRNA target genes were predicted using the TargetScan system (Release 7.0, http://www.targetscan.org/). The predicted genes were extracted and ranked by cumulative weighted context++ score [27] at the maximum of 1000 targets for each miRNA. To classify the target genes according to functional annotation, both gene ontology (GO) and pathway analyses were performed on the target genes of c-miRNA differentially expressed in the two groups based on the qRT-PCR results. In this study, the Database for Annotation, Visualization, and Integrated Discovery (DAVID) bioinformatic resources (version 6.7, http://david.abcc.ncifcrf.gov) [28] were applied to the potential target genes with *Bos taurus* set as the background species, to enrich characteristic GO terms and KEGG pathway defined by Kyoto Encyclopedia of Genes and Genomes (KEGG, http://www.genome.jp/kegg/) for the respective miRNA-mediated biological processes. Extraction of the terms was considered significant when the Benjamini *P*-value was < 0.10.

### Statistical analysis

The expression data are shown as means ± SDs and were compared between the feeding groups by the one-sided or two-sided Student’s *t*-test. It was expected that grazing would shift the muscle properties toward slow-oxidative type and decreased adipogenicity, which is associated with expression of miR-206 [29,30], miR-208b [31], and adipogenic genes such as *PPARG2* [20,21,25]. Based on this hypothesis, including presumptive declines in the expression of miR-196a, miR-885, *PPARG2*, *SCD1*, and *ELOVL6* by grazing, the significance of the differences in the *t*-test results for those targets was analyzed at one side. The effects of feeding condition were considered as significant if *P* < 0.05. Significance levels of *P* ≤ 0.10 were considered a statistical trend.

## Results

### Circulating miRNA profile of plasma extracellular vesicles in JB cattle

**Fig 1:**
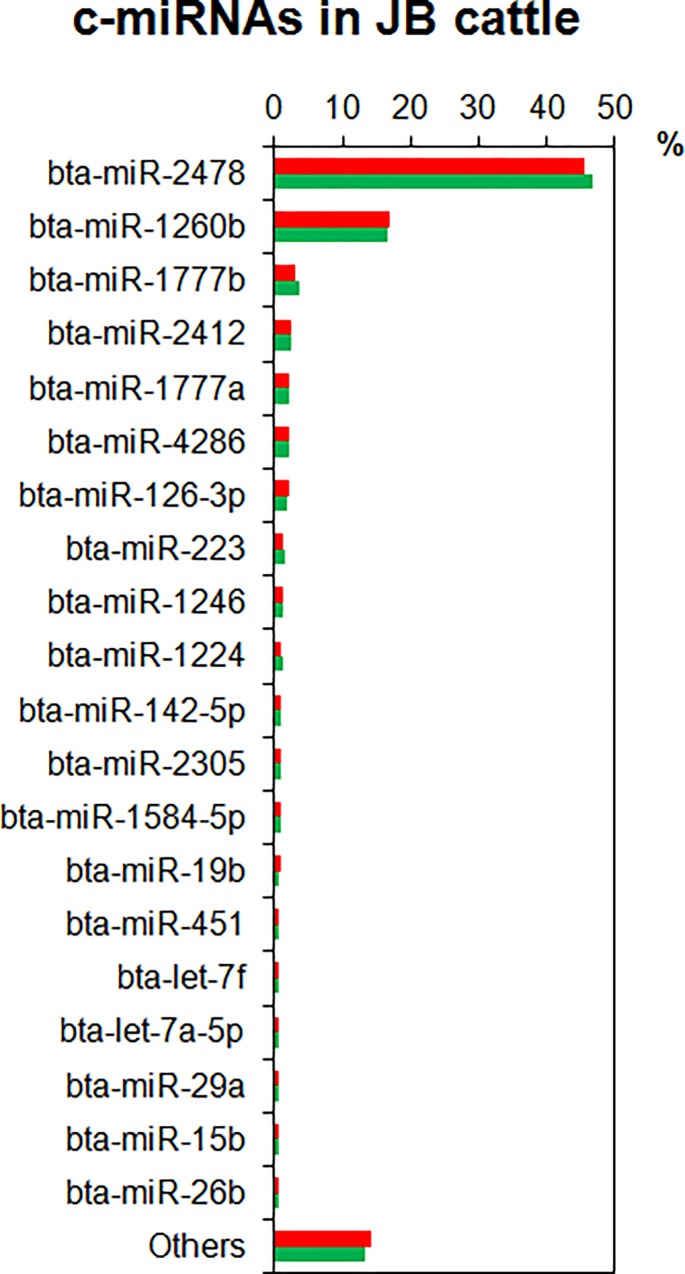
Circulating miRNA (c-miRNA) profiles in plasma extracellular vesicles of Japanese Black cattle obtained in microarray analysis. Percentages of c-miRNA contents in grain-fed (red bars) and grazing cattle (green bars) are indicated. The top 20 miRNAs in plasma of grazing cattle are listed.

To profile c-miRNAs of JB cattle fed under two different systems, we conducted microarray analysis using plasma EV precipitates prepared by ultracentrifugation. The c-miRNA profile obtained from the averaged raw signal value of microarray results for each group showed that the top 20 miRNAs were 85.7 and 86.8% of the total miRNAs in the grain-fed and grazing cattle, respectively (Fig 1). Most of the top 20 c-miRNAs did not differ between the groups in the order of the amount or the ratio of composition. The only differences were that miR-20a was present at higher levels in the grain-fed cattle than in the grazing cattle, and that miR-29a was present at higher levels in the grazing cattle, although both miR-20a and miR-29a were within the top 25 miRNAs in both cattle groups. In the plasma samples of both groups, miR-2478 and miR-1260b were approximately 46 and 17% of the total miRNAs, respectively (Fig 1). Our previous studies revealed that skeletal muscle-specific miRNAs, namely miR-1, miR-133a/b, miR-206, miR-208a/b, miR-496, and miR-499, were abundant in the muscles of JB cattle [4], whereas none of them was detected in the plasma profiles except for a modest miR-486 content [24]. In contrast, the plasma samples were enriched with moderate concentrations of miRNAs associated with adipose tissue, such as miR-15a, miR-19a/b, miR-21, miR-27b, miR-92a/b, miR-103, miR-106a/b, miR-107, miR-125b, and miR-150 [11,12]. It is especially notable that the bovine-specific miR-1584-5p, miR-2305, miR-2412, and miR-2478, most of which are associated with energy metabolism [32], were highly enriched in the plasma samples of both groups in the present study.

### Feeding conditions affect circulating miRNA contents in JB cattle

The statistical SAM analysis led to the extraction of a total of 15 c-miRNAs that differed between the groups (*P* < 0.05, Table 3). Of those miRNAs, the contents of miR-652, miR-30d, miR-301a, miR-345-5p, miR-374b, miR-425-5p, miR-23b-3p, miR-30b-5p, miR-17-5p, miR-98, miR-28, and miR-874 were lower in the plasma of the grazing cattle than in that of the grain-fed cattle, whereas the contents of miR-10b, miR-2368-3p, miR-885, and miR-2425-3p were higher in the plasma of the grazing cattle. The contents of miR-2368-3p and miR-2425-3p, putative ruminant-specific miRNAs [31], were very low (less than 0.067%), and no GO term was extracted from the predicted target genes of either miRNA by DAVID analysis. Therefore, we further focused on the other 13 miRNAs as biologically relevant c-miRNAs.

**Table 3:** Top 20 c-miRNAs considered as significantly different between feeding conditions of JB cattle.

miRNA	*P*-value	Ranking	Rank by fold change	log2(G/H)^1^
miR-652	0.010	1	109	-1.040
miR-30d	0.012	2	111	-0.893
miR-301a	0.016	3	6	-4.240
miR-345-5p	0.017	4	101	-1.323
miR-2368-3p	0.025	5	2	5.350
miR-374b	0.030	6	3	-4.653
miR-425-5p	0.031	7	131	-0.530
miR-885	0.035	8	1	5.587
miR-23b-3p	0.035	9	115	-0.707
miR-30b-5p	0.040	10	143	-0.413
miR-17-5p	0.040	11	137	-0.443
miR-98	0.042	12	8	-4.107
miR-2425-3p	0.045	13	90	1.590
miR-28	0.046	14	11	-3.917
miR-874	0.048	15	10	-3.927
miR-17-3p	0.055	16	13	-3.773
miR-10b	0.056	17	112	0.877
miR-151-3p	0.056	18	5	-4.327
miR-30e-5p	0.058	19	14	-3.740
miR-130b	0.059	20	4	-4.543

The data was statistically analyzed by SAM method as described in Materials and Methods.

^1^log2(G/H): the value of log2(grazing/grain-fed)

**Fig 2:**
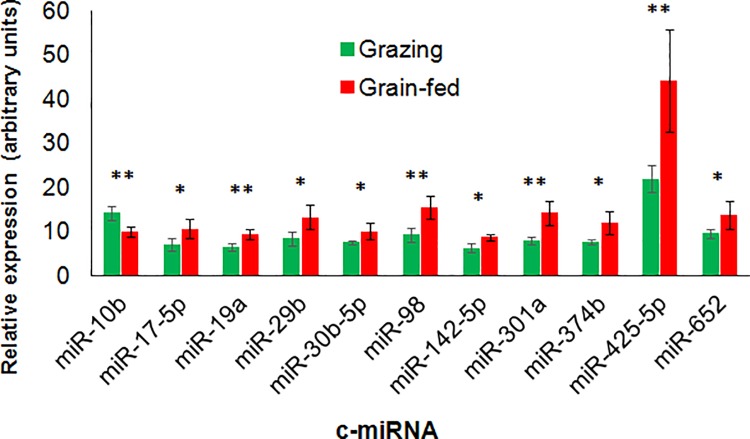
Circulating miRNA expression in plasma extracellular vesicles of grain-fed and grazing Japanese Black cattle analyzed by qRT-PCR. ** and * indicate the differences between the feeding conditions at *P* < 0.01 and < 0.05, respectively.

We then conducted qPCR for the contents of c-miRNAs of interest, not only to validate the results of microarray analysis for those 13 miRNAs and the miRNAs below the top 20 in SAM analysis (miR-29b, miR-197), but also to further explore potential feeding-induced miRNAs among adipose-enriched (miR-15a, miR-19a, miR-27b, miR-92a, miR-103, miR-142-5p), plasma-enriched (miR-2412, miR-2478), and bovine-specific (miR-2284x and miR-2295) miRNAs [31]. The results revealed that circulating miR-10b content was higher in grazing cattle than in grain-fed cattle (*P* = 0.005, Fig 2). In contrast, the grazing cattle showed significantly lower contents of miR-17-5p (*P* = 0.031), miR-19a (*P* = 0.007), miR-29b (*P* = 0.021), miR-30b-5p (*P* = 0.035), miR-98 (*P* = 0.006), miR-142-5p (*P* = 0.013), miR-301a (*P* = 0.005), miR-374b (*P* = 0.016), miR-425-5p (*P* = 0.010), and miR-652 (*P* = 0.046). All other miRNAs tested (miR-15a, miR-23b-3p, miR-27b, miR-30d-5p, miR-92a, miR-140, miR-197, miR-345-5p, miR-451, miR-885, miR-2284x, miR-2295, miR-2412, and miR-2478) were not differently expressed between the groups (*P* > 0.10), except that miR-103 content tended to be higher in the grain-fed cattle than in the grazing cattle (*P* = 0.057).

### Feeding conditions also affect skeletal muscle miRNA expression

Next, we analyzed the muscle tissue expression of the 11 miRNAs that differed significantly between the groups (miR-10b, miR-17-5p, miR-19a, miR-29b, miR-30b-5p, miR-98, miR-142-5p, miR-301a, miR-374b, miR-425-5p, and miR-652) to determine whether or not c-miRNA expression was associated with that of skeletal muscle tissue miRNAs.

**Fig 3:**
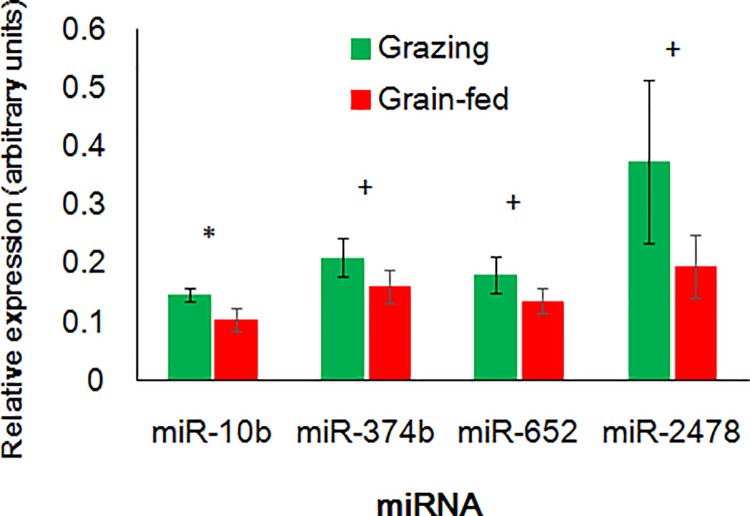
miRNA expression in *longissimus lumborum* muscle of grain-fed and grazing Japanese Black cattle analyzed by qRT-PCR. * and + indicate the differences between the feeding conditions at *P* < 0.05 and < 0.10, respectively.

Intriguingly, the miR-10b expression in LL muscle was coincidently higher in the grazing cattle than in the grain-fed cattle (*P* = 0.024, Fig 3). miR-374b and miR-652 also tended toward higher expression in the grazing cattle than in the grain-fed cattle (*P* = 0.062 and = 0.061, respectively). We also conducted qPCR of miRNAs that are enriched in muscle or plasma (miR-21-5p, miR-30d-5p, miR-103, miR-206, miR-208b, miR-451, miR-486, miR-499, miR-2412, and miR-2478), some of which are abundant in bovine skeletal muscles [4]. Of those miRNAs, only miR-2478 in LL muscle tended to differ between the feeding conditions (*P* = 0.053).

To predict potential cellular biological events that the significant miRNAs are involved in, a functional annotation of the miRNA target genes predicted by TargetScan was performed by DAVID. The results of the GO and KEGG pathway analyses showed that the potential target genes of uniquely upregulated miR-10b were associated with cell adhesion (Table 4). The potential targets of downregulated c-miRNAs were associated with protein phosphorylation and phosphate metabolism (miR-17-5p), endocytosis (miR-19a, miR-301a), mTOR signaling (miR-19a), MAPK signaling (miR-19a, miR-98), ECM-receptor interaction and adipocytokine signaling (miR-29b), skeletal muscle tissue development (miR-30b-5p), blood vessel development and morphogenesis, and regulation of actin cytoskeleton (miR-301a). The potential targets of the most plasma-enriched miR-2478 were associated with protein localization and transport. The genes predicted as targets of miR-374b and miR-652 were not significantly linked with any specific GO terms or KEGG pathways.

**Table 4:** Potential cellular biological events predicted from the microRNA target genes.

miRNA	Category	Term	Fold enrichment^1^	Benjamini
miR-10b				
	GOTERM_BP_FAT	GO:0007156~homophilic cell adhesion	4.535	0.003
	GOTERM_BP_FAT	GO:0016337~cell-cell adhesion	3.443	0.004
	GOTERM_BP_FAT	GO:0022610~biological adhesion	2.203	0.062
	GOTERM_BP_FAT	GO:0007155~cell adhesion	2.203	0.062
miR-17-5p				
	GOTERM_BP_FAT	GO:0006468~protein amino acid phosphorylation	2.161	0.010
	GOTERM_BP_FAT	GO:0045449~regulation of transcription	1.598	0.017
	GOTERM_BP_FAT	GO:0006796~phosphate metabolic process	1.836	0.021
	GOTERM_BP_FAT	GO:0006793~phosphorus metabolic process	1.836	0.021
	GOTERM_BP_FAT	GO:0016310~phosphorylation	1.905	0.035
	GOTERM_BP_FAT	GO:0010604~positive regulation of macromolecule metabolic process	2.142	0.084
	GOTERM_BP_FAT	GO:0007242~intracellular signaling cascade	1.847	0.097
	KEGG_PATHWAY	bta05219:Bladder cancer	5.341	0.075
miR-19a				
	GOTERM_BP_FAT	GO:0007242~intracellular signaling cascade	2.012	0.028
	GOTERM_BP_FAT	GO:0051094~positive regulation of developmental process	3.299	0.075
	KEGG_PATHWAY	bta04630:Jak-STAT signaling pathway	2.985	0.012
	KEGG_PATHWAY	bta05211:Renal cell carcinoma	3.817	0.033
	KEGG_PATHWAY	bta04930:Type II diabetes mellitus	4.297	0.049
	KEGG_PATHWAY	bta04144:Endocytosis	2.268	0.058
	KEGG_PATHWAY	bta04010:MAPK signaling pathway	2.019	0.066
	KEGG_PATHWAY	bta04150:mTOR signaling pathway	3.981	0.071
	KEGG_PATHWAY	bta05410:Hypertrophic cardiomyopathy (HCM)	3.133	0.079
miR-29b				
	KEGG_PATHWAY	bta04510:Focal adhesion	3.529	0.000
	KEGG_PATHWAY	bta04512:ECM-receptor interaction	4.611	0.001
	KEGG_PATHWAY	bta05200:Pathways in cancer	2.201	0.008
	KEGG_PATHWAY	bta05222:Small cell lung cancer	3.705	0.011
	KEGG_PATHWAY	bta04920:Adipocytokine signaling pathway	3.529	0.097
miR-30b-5p				
	GOTERM_BP_FAT	GO:0007242~intracellular signaling cascade	1.996	0.034
	GOTERM_BP_FAT	GO:0007519~skeletal muscle tissue development	5.780	0.098
	GOTERM_BP_FAT	GO:0060538~skeletal muscle organ development	5.780	0.098
miR-98				
	KEGG_PATHWAY	bta04010:MAPK signaling pathway	2.598	< 0.001
	KEGG_PATHWAY	bta05200:Pathways in cancer	2.203	0.005
miR-142-5p				
	KEGG_PATHWAY	bta05200:Pathways in cancer	2.106	0.048
miR-301a				
	GOTERM_BP_FAT	GO:0007242~intracellular signaling cascade	2.211	0.001
	GOTERM_BP_FAT	GO:0048514~blood vessel morphogenesis	3.627	0.051
	GOTERM_BP_FAT	GO:0044087~regulation of cellular component biogenesis	4.885	0.073
	GOTERM_BP_FAT	GO:0007264~small GTPase mediated signal transduction	2.675	0.074
	GOTERM_BP_FAT	GO:0008104~protein localization	1.932	0.076
	GOTERM_BP_FAT	GO:0009792~embryonic development ending in birth or egg hatching	2.643	0.079
	GOTERM_BP_FAT	GO:0043009~chordate embryonic development	2.660	0.081
	GOTERM_BP_FAT	GO:0001568~blood vessel development	3.043	0.085
	GOTERM_BP_FAT	GO:0051173~positive regulation of nitrogen compound metabolic process	2.333	0.085
	GOTERM_BP_FAT	GO:0001944~vasculature development	3.165	0.086
	GOTERM_BP_FAT	GO:0045935~positive regulation of nucleobase, nucleoside, nucleotide and nucleic acid metabolic process	2.300	0.091
	GOTERM_BP_FAT	GO:0010628~positive regulation of gene expression	2.338	0.094
	KEGG_PATHWAY	bta04144:Endocytosis	2.784	0.003
	KEGG_PATHWAY	bta04115:p53 signaling pathway	4.262	0.006
	KEGG_PATHWAY	bta04810:Regulation of actin cytoskeleton	2.281	0.048
miR-2478				
	GOTERM_BP_FAT	GO:0045184~establishment of protein localization	2.096	0.055
	GOTERM_BP_FAT	GO:0015031~protein transport	2.101	0.079
	GOTERM_BP_FAT	GO:0008104~protein localization	2.077	0.088
	KEGG_PATHWAY	bta04340:Hedgehog signaling pathway	4.629	0.079

The data were statistically analyzed by DAVID as described in Materials and Methods.

^1^Fold enrichment = (genes in list that are annotated with the term)/(total numbers of genes that are annotated with any term in the term category)

### Association between miRNA target expression and miRNAs in skeletal muscle

**Fig 4:**
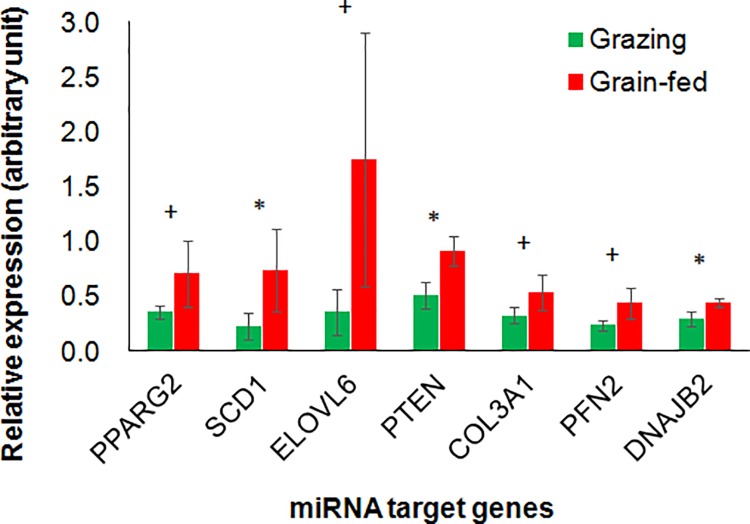
miRNA target gene expression in *longissimus lumborum* muscle of grain-fed and grazing Japanese Black cattle analyzed by qRT-PCR. * and + indicate the differences between the feeding conditions at *P* < 0.05 and < 0.10, respectively.

Hypothesizing that the differences in miRNA expression could affect the regulation of miRNA target genes, we further analyzed expression of potential miRNA target genes in LL muscle tissue by qPCR. Although cell adhesion-related genes were employed for extraction of miR-10b-related GO terms in bioinformatic analysis, those genes showed lower cumulative weighed context++ scores [27] than *PTEN* and *ELOVL6* genes. In addition, most of the cell adhesion-related genes were not expressed in JB cattle muscles according to our preliminary analysis. Therefore, based on the result of the target gene prediction by TargetScan analysis, we especially focused on the genes associated with fat and muscle growth, development, and/or metabolism among the predicted genes, to assess the relationships between circulating or muscle miRNAs and the gene expression related to beef quality. One of the lipogenic genes, *SCD1*, which is predicted as a target of miR-98, miR-142-5p, and miR-2478, showed higher expression in the grain-fed than in the grazing cattle (*P* = 0.038, Fig 4). The other adipogenic or lipogenic genes, miR-301a target *PPARG2* and a target of miR-10b and miR-2478, *ELOVL6*, also tended to differ between the groups (*P* = 0.058 and = 0.051, respectively). The expression of *PTEN*, the potential target of miR-10b, miR-17-5p, miR-19a, miR-29b, miR-30b-5p, miR-142-5p, miR-301a, miR-652, and miR-2478, was lower in the grazing cattle than in the grain-fed cattle (*P* = 0.011). The mRNA expression of actin-binding protein *PFN2*, the potential target of miR-17-5p, miR-19a, miR-30b-5p, miR-142-5p, miR-301a, and miR-2478, also tended to be downregulated in the grazing cattle compared to the grain-fed cattle (*P* = 0.064), as did the potential miR-29b targets *DNAJB2* (*P* = 0.021) and *COL3A1* (*P* = 0.100). These expression patterns of the miRNA targets indicated that the expressions of these potential miRNA target genes are associated with grain-fed housing-induced changes in circulating and muscle miRNAs in JB cattle. These relevant results are summarized in Table 5.

**Table 5:** Summary of altered miRNAs and the miRtarget genes of interest in LL muscle of the grazing JB cattle compared to the grain-fed.

			Target gene^1^						
				DNAJB2	SCD1	EVOLV6	PPARG	PTEN	PFN2
			Category	heat shock proteins	adipogenesis	adipogenesis	adipogenesis	PI3K signaling	actin-binding
miRNA changes			LL muscle^2^	**↓↓**	**↓↓**	**↓**	**↓**	**↓↓**	**↓**
miRNA	Circulation^2^	LL muscle^2^							
miR-10b	**↑↑**	**↑↑**				+		+	
miR-17-5p	**↓↓**	**-**						+	+
miR-19a	**↓↓**	**-**						+	+
miR-29b	**↓↓**	**-**		+				+	
miR-30b-5p	**↓↓**	**-**						+	+
miR-98	**↓↓**	**-**			+				
miR-142-5p	**↓↓**	**-**			+			+	+
miR-301a	**↓↓**	**-**					+	+	+
miR-374b	**↓↓**	**↑**							
miR-425-5p	**↓↓**	**-**							
miR-652	**↓↓**	**↑**		+				+	
miR-2478	**-**	**↑**		+	+	+		+	+

^1^+ indicates the gene targeted by the altered miRNAs in the top row of this table.

^2^↑↑or↓↓: up- or down-regulated at *P* < 0.05, ↑or↓: up- or down-regulated at *P* < 0.10, -: not changed (*P* ≥ 0.10).

## Discussion

It has been demonstrated that diet and supplementary nutrients affect miRNA expression in adipose tissues of mice [14–16], sheep [17] and cattle [18]. However, miRNAs in circulation and skeletal muscle have poorly understood in farm animals thus far. Nevertheless, a polyunsaturated fatty acids (PUFAs)-enriched diet might have the potential to alter the expression of c-miRNAs linked to changes in dietary fatty acids (as inferred from plasma fatty acid concentration) [33]. In the present study, we focused on the effects of feeding conditions on c-miRNAs and skeletal muscle miRNAs as well as on muscle target genes in JB cattle, whose beef has high marbling potential, to understand not only the implications of feeding diet and grazing on the expression of the set of miRNAs and their target genes in skeletal muscles, but also the associations between altered muscle gene expression and c-miRNAs. Most of studies examining effects of grain-rich high concentrate feeding compared to high roughage feeding or grazing resulted in higher IMF depot but accompanied higher body weight of JB cattle in the concentrate feeding than in the roughage feeding [22,34], nevertheless a high concentrate feeding that showed higher IMF depot without significant difference in final body weight between cattle fed high and low concentrate indicated a positive effect of grain-rich concentrate [21,35]. In the present study, grain-fed cattle showed significantly higher body weight than grazing cattle, nevertheless the higher lipogenic gene expression in the grain-fed cattle could partly be caused by the difference in the diet.

### Characteristics of plasma extracellular vesicle miRNA profile in JB cattle

We profiled the plasma EV miRNAs in JB cattle in both the indoor grain-fed and grazing conditions. Previously we successfully prepared EV miRNAs from plasma of cattle by an ultra-centrifugation method [24], and we therefore employed the same method in the present study. The present results revealed no significant difference between the feeding conditions in the profiles of abundant miRNAs in the plasma EV of cattle. Those profiles were enriched with bovine-specific miR-1584-5p, miR-2305, miR-2412, and miR-2478, which are associated with energy metabolism [32]. Although it is unknown why those miRNAs were abundant in the profiles, they may be involved in bovine-specific energy metabolism. Regarding the top 20 c-miRNAs, the c-miRNA profiles of both JB cattle groups were considerably similar to those of JSH cattle [24], except that in the latter, miR-15b, miR-19b, miR-20a, and miR-26a were not in the top 20 but were above 30. The differences between JB and JSH in beef quality traits such as marbling (IMF depot) [36] may not be associated with composition of those major c-miRNAs.

### miR-10b and miR-2478 in circulation and skeletal muscle of JB cattle

The present results of microarray and qPCR revealed that the grazing JB cattle had a higher plasma content of miR-10b and lower plasma contents of miR-17-5p, miR-23b-3p, miR-28, miR-30b-5p, miR-30d, miR-98, miR-301a, miR-345-5p, miR-374b, miR-425-5p, miR-652, and miR-874 than the grain-fed cattle. Most of the differently expressed c-miRNAs were downregulated in the grazing group. In contrast, the unique miR-10b upregulation in the grazing cattle plasma deserves taking notice. As we hypothesize that c-miRNA changes indicate alterations of skeletal muscle physiology, miR-10b expression in the LL muscle was upregulated coincidently with the change of circulating miR-10b, suggesting involvement of miRNAs in muscle property alteration. The expression levels of muscle miR-374b, miR-652, and miR-2478 also tended to be higher in the LL muscle of the grazing cattle. To date, c-miRNAs in cattle have been reported in relation to grazing [24], heat stress [23], pregnancy [37], and viral infection [38], but not in relation to changes in circulating miR-10b. The altered miRNAs could be indicators of feeding conditions such as grain abundance and may be useful noninvasive biomarkers to monitor feeding.

### Association of miR-10b with the target *PTEN* in JB cattle muscle

TargetScan analysis predicted that miR-10b and miR-2478 targeted *PTEN*. According to a bioinformatic analysis of potential implications of the circulating and muscle miR-10b upregulation, GO terms related to cell adhesion were extracted as biological events associated with the predicted miR-10b target genes. However, most of the cell adhesion-related genes might not be meaningful due to lack of expression in JB cattle muscle in our preliminary result. The *PTEN* expression in the LL muscle was lower in the grazing JB cattle than in the grain-fed cattle. Indeed, it was demonstrated that miR-10b can target *PTEN* and cause cell proliferation and/or migration [39,40]. PTEN is the phosphatase that converts phosphatidylinositol 3,4,5-triphosphate (PIP_3_) to inactivate phosphatidylinositol 4,5-bisphosphate (PI-4,5-P_2_), and it inhibits phosphatidylinositol-3-kinase/Akt (PI3K/Akt) signaling [41], which is a key determinant of the insulin-dependent increase in glucose uptake into muscle and adipose cells. Overexpression of *PTEN* inhibits GLUT4 translocation and glucose uptake in 3T3-L1 adipocytes [42]. Skeletal muscle-specific deletion of *PTEN* protects mice from developing insulin resistance and diabetes induced by a high-fat diet, and thus improves glucose homeostasis [43]. By inhibiting PI3K/Akt signaling, PTEN can regulate muscle cell differentiation [44] and hypertrophy under the reduced availability of insulin-like growth factor binding protein 2 (IGFBP2) [45]. Thus, the reduced *PTEN* expression in the grazing cattle suggests alterations in both muscle cell growth and glucose homeostasis in the LL muscle, which might be modulated by miR-10b and possibly miR-2478, and might further affect final beef production.

### Potential impact of miR-2478 and its targets on JB cattle muscle

miR-2478 potentially targets mRNAs of lipogenic genes *ELOVL6* and *SCD1*. ELOVL6 catalyzes the elongation of saturated and monounsaturated fatty acids with 12-, 14-, and 16-carbons in the endoplasmic reticulum of the adipocyte. SCD1 converts a portion of stearic acid (18:0) into oleic acid (18:1) [46]. The expression of both *ELOVL6* and *SCD1* increased during IMF deposition and temporally had a very large upregulation in *longissimus* muscle of Angus steers fed a high-starch diet after an early weaning, compared to normally weaned cattle [47]. This supports our present observation of downregulated *ELOVL6* and *SCD1* expression in the grazing JB cattle compared to the grain-fed cattle. The downregulated *ELOVL6* and *SCD1* expression in our study was associated with the increased miR-10b and miR-2478 expression in the LL muscle of grazing JB cattle. Therefore, it is likely that the expression of those lipogenic genes induced by grain-rich feeding was also modulated by miR-10b and/or miR-2478 in the IMF of the LL muscle, and might affect beef quality.

miR-2478 was also predicted to target *PFN2* and *DNAJB2*, whose expression tended to be reduced in the LL muscle of the grazing cattle in the present study. Especially, profilin binds to actin filament at the barbed end and affects actin homeostasis during cell movement [48]. In addition, because profilin can bind to PI-4,5-P_2_ [49], *PFN2* expression might also be associated with the alteration of *PTEN* expression in the LL muscle, since profilin overexpression induced *PTEN* upregulation and reduced Akt expression [50]. Expression of DNAJB2, a co-chaperone regulator of heat shock protein Hsp70 that is expressed principally in the nervous system, is induced in human skeletal muscle during its recovery from damage by exercise [51]. In a study using samples of skeletal muscle and the pathologies associated with protein aggregation, it was proposed that DNAJB2 has a role in protein turnover in skeletal muscle [52]. The higher muscle *DNAJB2* expression in the grain-fed JB cattle than in the grazing cattle might indicate that *DNAJB2* was upregulated via downregulation of miR-2478 by some cellular stress due to difference between the feeding conditions.

### Association of circulating miRNA with muscle miRNA and the biological impact on JB cattle

In this study, it is concluded that the differing c-miRNAs between the feeding conditions could be an indicator not only of the grain abundance of a diet but also changes in gene expression associated with PI3K signaling and lipogenesis in the LL muscle of JB cattle. To date, however, the effects of c-miRNAs on tissues including EV recipient cells remain poorly understood.

We concluded that difference in circulating miR-10b between the feeding conditions was associated with the miR-10b upregulation in the LL muscle of cattle. EVs containing miR-210 isolated from metastatic breast cancer cells promote metastasis via the induction of angiogenesis in the tumor, and the addition of miR-210-enriched EVs induced the activation of endothelial cells in vitro [53]. Although roles of c-miRNAs to downregulate the muscle target genes were not demonstrated in this study, at least c-miRNAs, especially miR-10b, might either be taken into the LL muscle, or be secreted from the tissues inside the cattle body including muscles. High-fat diet feeding to cattle raises expression of miRNAs including miR-19a in both the subcutaneous and visceral fat [18], suggesting that high energy diet cause increase in adipose tissue miRNA expression. It is therefore likely that the present grain feeding as a higher energy diet altered miRNA expression in adipose tissue and might promote secretion of exosomes containing miRNAs such as miR-19a into plasma of the grain-fed cattle. It is necessary to clarify the departing and destination tissues of c-miRNAs and the mechanism how the c-miRNAs are balanced between circulation and various tissues of secretion and recipient.

In the present study, muscle *PTEN* and *PFN2* were the predicted targets of most of the altered c-miRNAs (miR-17-5p, miR-19a, miR-30b-5p, miR-142-5p, miR-301a) between the feeding conditions, as well as miR-10b. Moreover, the results of bioinformatic functional annotation indicated that the altered c-miRNAs are associated with cellular biological events involved in phosphorylation, various intracellular signaling pathways, skeletal muscle and blood vessel development, and protein localization. Taking together, c-miRNAs might affect tissue development and maturation in cattle. Further research is needed to elucidate actions of feeding-induced c-miRNAs on the expression of the target genes in potential recipient tissues.

## Conclusion

The present study revealed that the grazing of JB cattle on pasture affected the plasma c-miRNAs compared to grain-feeding JB cattle. Especially, miR-10b expression increased in both circulation and the LL muscle in the grazing cattle. The altered expression of miR-10b and miR-2478 in the LL muscle was further associated with the expression of the target lipogenic genes, *ELOVL6* and *SCD1*, respectively. Moreover, expression of the factor regulating glucose homeostasis, *PTEN*, was also reduced in the grazing cattle. These results indicated that changes in c-miRNAs induced by a grain-rich diet are associated with skeletal muscle miRNA and the potential target gene expression, suggesting in turn that muscle miRNAs are involved in the regulation of muscle and adipogenic genes that determine beef quality.

## References

[1] Bartel DP (2004) MicroRNAs: genomics, biogenesis, mechanism, and function. Cell 116: 281–297. 14744438 10.1016/s0092-8674(04)00045-5

[2] Lewis BP, Shih IH, Jones-Rhoades MW, Bartel DP, Burge CB (2003) Prediction of mammalian microRNA targets. Cell 115: 787–798. 14697198 10.1016/s0092-8674(03)01018-3

[3] Liu Y, Li M, Ma J, Zhang J, Zhou C, et al (2013) Identification of differences in microRNA transcriptomes between porcine oxidative and glycolytic skeletal muscles. BMC Mol Biol 14: 7 10.1186/1471-2199-14-7 23419046 PMC3599761

[4] Muroya S, Taniguchi M, Shibata M, Oe M, Ojima K, et al (2013) Profiling of differentially expressed microRNA and the bioinformatic target gene analyses in bovine fast- and slow-type muscles by massively parallel sequencing. J Anim Sci 91: 90–103. 10.2527/jas.2012-5371 23100578

[5] Li HY, Xi QY, Xiong YY, Liu XL, Cheng X, et al (2012) Identification and comparison of microRNAs from skeletal muscle and adipose tissues from two porcine breeds. Anim Genet 43: 704–713. 10.1111/j.1365-2052.2012.02332.x 22497549

[6] Sun J, Zhang B, Lan X, Zhang C, Lei C, et al (2014) Comparative transcriptome analysis reveals significant differences in MicroRNA expression and their target genes between adipose and muscular tissues in cattle. PLoS One 9: e102142 10.1371/journal.pone.0102142 25006962 PMC4090223

[7] Horikawa A, Ogasawara H, Okada K, Kobayashi M, M H (2015) Grazing-induced changes in muscle microRNA-206 and -208b expression in association with myogenic gene expression in cattle. Animal Science Journal in press. 10.1111/asj.12381 26122272

[8] Muroya S, Oe M, Nakajima I, Ojima K, Chikuni K (2014) CE-TOF MS-based metabolomic profiling revealed characteristic metabolic pathways in postmortem porcine fast and slow type muscles. Meat Sci 98: 726–735. 10.1016/j.meatsci.2014.07.018 25105492

[9] Ryu YC, Kim BC (2005) The relationship between muscle fiber characteristics, postmortem metabolic rate, and meat quality of pig longissimus dorsi muscle. Meat Sci 71: 351–357. 10.1016/j.meatsci.2005.04.015 22064236

[10] Choi YM, Kim BC (2009) Muscle fiber characteristics, myofibrillar protein isoforms, and meat quality. Livestock Science 122: 105–118.

[11] Kim JM, Lim KS, Hong JS, Kang JH, Lee YS, et al (2015) A polymorphism in the porcine miR-208b is associated with microRNA biogenesis and expressions of SOX-6 and MYH7 with effects on muscle fibre characteristics and meat quality. Anim Genet 46: 73–77. 10.1111/age.12255 25530254

[12] Ponsuksili S, Du Y, Hadlich F, Siengdee P, Murani E, et al (2013) Correlated mRNAs and miRNAs from co-expression and regulatory networks affect porcine muscle and finally meat properties. BMC Genomics 14: 533 10.1186/1471-2164-14-533 23915301 PMC3750351

[13] Shen L, Chen L, Zhang S, Zhang Y, Wang J, et al (2016) MicroRNA-23a reduces slow myosin heavy chain isoforms composition through myocyte enhancer factor 2C (MEF2C) and potentially influences meat quality. Meat Sci 116: 201–206. 10.1016/j.meatsci.2016.02.023 26897085

[14] Takanabe R, Ono K, Abe Y, Takaya T, Horie T, et al (2008) Up-regulated expression of microRNA-143 in association with obesity in adipose tissue of mice fed high-fat diet. Biochem Biophys Res Commun 376: 728–732. 10.1016/j.bbrc.2008.09.050 18809385

[15] Parra P, Serra F, Palou A (2010) Expression of adipose microRNAs is sensitive to dietary conjugated linoleic acid treatment in mice. PLoS One 5: e13005 10.1371/journal.pone.0013005 20886002 PMC2946340

[16] Chartoumpekis DV, Zaravinos A, Ziros PG, Iskrenova RP, Psyrogiannis AI, et al (2012) Differential expression of microRNAs in adipose tissue after long-term high-fat diet-induced obesity in mice. PLoS One 7: e34872 10.1371/journal.pone.0034872 22496873 PMC3319598

[17] Meale SJ, Romao JM, He ML, Chaves AV, McAllister TA, et al (2014) Effect of diet on microRNA expression in ovine subcutaneous and visceral adipose tissues. J Anim Sci 92: 3328–3337. 10.2527/jas.2014-7710 24893997

[18] Romao JM, Jin W, He M, McAllister T, Guan LL (2012) Altered microRNA expression in bovine subcutaneous and visceral adipose tissues from cattle under different diet. PLoS One 7: e40605 10.1371/journal.pone.0040605 22815773 PMC3398999

[19] Liu J, Du X, Zhou J, Pan Z, Liu H, et al (2014) MicroRNA-26b functions as a proapoptotic factor in porcine follicular Granulosa cells by targeting Sma-and Mad-related protein 4. Biol Reprod 91: 146 10.1095/biolreprod.114.122788 25395673

[20] Shibata M, Matsumoto K, Oe M, Ohnishi-Kameyama M, Ojima K, et al (2009) Differential expression of the skeletal muscle proteome in grazed cattle. J Anim Sci 87: 2700–2708. 10.2527/jas.2008-1486 19420231

[21] Yamada T, Nakanishi N (2012) Effects of the roughage/concentrate ratio on the expression of angiogenic growth factors in adipose tissue of fattening Wagyu steers. Meat Sci 90: 807–813. 10.1016/j.meatsci.2011.11.019 22133587

[22] Shibata MMK, Hikino Y, Yamamoto N (2014) Effect of Indoor Concentrate Feeding vs. Outdoor Grazing on the Expression of Genes Involved in Muscle Growth and Nutrient Content in Japanese Black Steer Muscle. Scientific Research 4: 297–304.

[23] Zheng Y, Chen KL, Zheng XM, Li HX, Wang GL (2014) Identification and bioinformatics analysis of microRNAs associated with stress and immune response in serum of heat-stressed and normal Holstein cows. Cell Stress Chaperones 19: 973–981. 10.1007/s12192-014-0521-8 24917036 PMC4389857

[24] Muroya S, Ogasawara H, Hojito M (2015) Grazing Affects Exosomal Circulating MicroRNAs in Cattle. PLoS One 10: e0136475 10.1371/journal.pone.0136475 26308447 PMC4550388

[25] Shibata M, Hikino Y, Imanari M, Matsumoto K, Yamamoto N (2015) Influence of rice whole-crop silage diet on growth performance, carcass and meat characteristics and muscle-related gene expression in Japanese Black steers. Animal Science Journal 87: 929–937. 10.1111/asj.12519 26420580

[26] Tusher VG, Tibshirani R, Chu G (2001) Significance analysis of microarrays applied to the ionizing radiation response. Proc Natl Acad Sci U S A 98: 5116–5121. 11309499 10.1073/pnas.091062498 PMC33173

[27] Agarwal V, Bell GW, Nam JW, Bartel DP (2015) Predicting effective microRNA target sites in mammalian mRNAs. Elife 4. 10.7554/eLife.05005 PMC4532895 26267216

[28] Huang da W, Sherman BT, Lempicki RA (2009) Bioinformatics enrichment tools: paths toward the comprehensive functional analysis of large gene lists. Nucleic Acids Res 37: 1–13. 10.1093/nar/gkn923 19033363 PMC2615629

[29] Jeng SF, Rau CS, Liliang PC, Wu CJ, Lu TH, et al (2009) Profiling muscle-specific microRNA expression after peripheral denervation and reinnervation in a rat model. J Neurotrauma 26: 2345–2353. 10.1089/neu.2009.0960 19586368

[30] Endo K, Weng H, Naito Y, Sasaoka T, Takahashi A, et al (2013) Classification of various muscular tissues using miRNA profiling. Biomed Res 34: 289–299. 24389405 10.2220/biomedres.34.289

[31] van Rooij E, Quiat D, Johnson BA, Sutherland LB, Qi X, et al (2009) A family of microRNAs encoded by myosin genes governs myosin expression and muscle performance. Dev Cell 17: 662–673. 10.1016/j.devcel.2009.10.013 19922871 PMC2796371

[32] Romao JM, Jin W, He M, McAllister T, Guan lL (2014) MicroRNAs in bovine adipogenesis: genomic context, expression and function. BMC Genomics 15: 137 10.1186/1471-2164-15-137 24548287 PMC3930007

[33] Ortega FJ, Cardona-Alvarado MI, Mercader JM, Moreno-Navarrete JM, Moreno M, et al (2015) Circulating profiling reveals the effect of a polyunsaturated fatty acid-enriched diet on common microRNAs. J Nutr Biochem 26: 1095–1101. 10.1016/j.jnutbio.2015.05.001 26092372

[34] Shibata M, Matsumoto K, Hikino Y, Oe M, Ojima K, et al (2011) Influence of different feeding systems on the growth performance and muscle development of Japanese Black steers. Meat Sci 89: 451–456. 10.1016/j.meatsci.2011.05.006 21641731

[35] Chung KY, Lunt DK, Kawachi H, Yano H, Smith SB (2007) Lipogenesis and stearoyl-CoA desaturase gene expression and enzyme activity in adipose tissue of short- and long-fed Angus and Wagyu steers fed corn- or hay-based diets. J Anim Sci 85: 380–387. 17235023 10.2527/jas.2006-087

[36] Zembayashi M, Lunt DK (1995) Distribution of intramuscular lipid throughout M. longissimus thoracis et lumborum in Japanese Black, Japanese Shorthorn, Holstein and Japanese Black crossbreds. Meat Sci 40: 211–216. 22059973 10.1016/0309-1740(94)00046-a

[37] Ioannidis J, Donadeu FX (2016) Circulating miRNA signatures of early pregnancy in cattle. BMC Genomics 17: 184 10.1186/s12864-016-2529-1 26939708 PMC4778341

[38] Farrell D, Shaughnessy RG, Britton L, MacHugh DE, Markey B, et al (2015) The Identification of Circulating MiRNA in Bovine Serum and Their Potential as Novel Biomarkers of Early Mycobacterium avium subsp paratuberculosis Infection. PLoS One 10: e0134310 10.1371/journal.pone.0134310 26218736 PMC4517789

[39] Mussnich P, D'Angelo D, Leone V, Croce CM, Fusco A (2013) The High Mobility Group A proteins contribute to thyroid cell transformation by regulating miR-603 and miR-10b expression. Mol Oncol 7: 531–542. 10.1016/j.molonc.2013.01.002 23384558 PMC5528467

[40] Bai W, Chen Y, Yang J, Niu P, Tian L, et al (2014) Aberrant miRNA profiles associated with chronic benzene poisoning. Exp Mol Pathol 96: 426–430. 10.1016/j.yexmp.2014.04.011 24780745

[41] Hu Z, Wang H, Lee IH, Modi S, Wang X, et al (2010) PTEN inhibition improves muscle regeneration in mice fed a high-fat diet. Diabetes 59: 1312–1320. 10.2337/db09-1155 20200318 PMC2874691

[42] Nakashima N, Sharma PM, Imamura T, Bookstein R, Olefsky JM (2000) The tumor suppressor PTEN negatively regulates insulin signaling in 3T3-L1 adipocytes. J Biol Chem 275: 12889–12895. 10777587 10.1074/jbc.275.17.12889

[43] Wijesekara N, Konrad D, Eweida M, Jefferies C, Liadis N, et al (2005) Muscle-specific Pten deletion protects against insulin resistance and diabetes. Mol Cell Biol 25: 1135–1145. 15657439 10.1128/MCB.25.3.1135-1145.2005 PMC544010

[44] Mandl A, Sarkes D, Carricaburu V, Jung V, Rameh L (2007) Serum withdrawal-induced accumulation of phosphoinositide 3-kinase lipids in differentiating 3T3-L6 myoblasts: distinct roles for Ship2 and PTEN. Mol Cell Biol 27: 8098–8112. 17893321 10.1128/MCB.00756-07 PMC2169165

[45] Sharples AP, Al-Shanti N, Hughes DC, Lewis MP, Stewart CE (2013) The role of insulin-like-growth factor binding protein 2 (IGFBP2) and phosphatase and tensin homologue (PTEN) in the regulation of myoblast differentiation and hypertrophy. Growth Horm IGF Res 23: 53–61. 10.1016/j.ghir.2013.03.004 23583027

[46] Waters SM, Kelly JP, O'Boyle P, Moloney AP, Kenny DA (2009) Effect of level and duration of dietary n-3 polyunsaturated fatty acid supplementation on the transcriptional regulation of Delta9-desaturase in muscle of beef cattle. J Anim Sci 87: 244–252. 10.2527/jas.2008-1005 18791145

[47] Moisa SJ, Shike DW, Faulkner DB, Meteer WT, Keisler D, et al (2014) Central Role of the PPARgamma Gene Network in Coordinating Beef Cattle Intramuscular Adipogenesis in Response to Weaning Age and Nutrition. Gene Regul Syst Bio 8: 17–32. 10.4137/GRSB.S11782 24516329 PMC3894150

[48] Pernier J, Shekhar S, Jegou A, Guichard B, Carlier MF (2016) Profilin Interaction with Actin Filament Barbed End Controls Dynamic Instability, Capping, Branching, and Motility. Dev Cell 36: 201–214. 10.1016/j.devcel.2015.12.024 26812019 PMC4729542

[49] Lambrechts A, van Damme J, Goethals M, Vandekerckhove J, Ampe C (1995) Purification and characterization of bovine profilin II. Actin, poly(L-proline) and inositolphospholipid binding. Eur J Biochem 230: 281–286. 7601111

[50] Das T, Bae YH, Wells A, Roy P (2009) Profilin-1 overexpression upregulates PTEN and suppresses AKT activation in breast cancer cells. J Cell Physiol 218: 436–443. 10.1002/jcp.21618 18937284 PMC2874249

[51] Mahoney DJ, Safdar A, Parise G, Melov S, Fu M, et al (2008) Gene expression profiling in human skeletal muscle during recovery from eccentric exercise. Am J Physiol Regul Integr Comp Physiol 294: R1901–1910. 10.1152/ajpregu.00847.2007 18321953 PMC2707850

[52] Claeys KG, Sozanska M, Martin JJ, Lacene E, Vignaud L, et al (2010) DNAJB2 expression in normal and diseased human and mouse skeletal muscle. Am J Pathol 176: 2901–2910. 10.2353/ajpath.2010.090663 20395441 PMC2877851

[53] Kosaka N, Iguchi H, Hagiwara K, Yoshioka Y, Takeshita F, et al (2013) Neutral sphingomyelinase 2 (nSMase2)-dependent exosomal transfer of angiogenic microRNAs regulate cancer cell metastasis. J Biol Chem 288: 10849–10859. 10.1074/jbc.M112.446831 23439645 PMC3624465

